# Pulsed Electric Fields Induce STING Palmitoylation and Polymerization Independently of Plasmid DNA Electrotransfer

**DOI:** 10.3390/pharmaceutics16030363

**Published:** 2024-03-05

**Authors:** Amanda Sales Conniff, Julie Singh, Richard Heller, Loree C. Heller

**Affiliations:** Department of Medical Engineering, University of South Florida, Tampa, FL 33612, USA; amandasales@usf.edu (A.S.C.); juliesingh@usf.edu (J.S.); rheller@usf.edu (R.H.)

**Keywords:** skeletal muscle, STING, palmitoylation, pulsed electric fields, electroporation, electrotransfer

## Abstract

Gene therapy approaches may target skeletal muscle due to its high protein-expressing nature and vascularization. Intramuscular plasmid DNA (pDNA) delivery via pulsed electric fields (PEFs) can be termed electroporation or electrotransfer. Nonviral delivery of plasmids to cells and tissues activates DNA-sensing pathways. The central signaling complex in cytosolic DNA sensing is the cyclic GMP-AMP synthase-stimulator of interferon genes (cGAS-STING). The effects of pDNA electrotransfer on the signaling of STING, a key adapter protein, remain incompletely characterized. STING undergoes several post-translational modifications which modulate its function, including palmitoylation. This study demonstrated that in mouse skeletal muscle, STING was constitutively palmitoylated at two sites, while an additional site was modified following electroporation independent of the presence of pDNA. This third palmitoylation site correlated with STING polymerization but not with STING activation. Expression of several palmitoyl acyltransferases, including zinc finger and DHHC motif containing 1 (zDHHC1), coincided with STING activation. Expression of several depalmitoylases, including palmitoyl protein thioesterase 2 (PPT2), was diminished in all PEF application groups. Therefore, STING may not be regulated by active modification by palmitate after electroporation but inversely by the downregulation of palmitate removal. These findings unveil intricate molecular changes induced by PEF application.

## 1. Introduction

The molecular mechanisms activated by pulsed electric fields (PEFs) whether alone or combined with molecular delivery are not fully understood. Myoblasts and skeletal muscle, like most cells and tissues, respond to electroporation with changes in gene and protein expression [1,2,3,4,5], as well as direct physical effects. These cells and tissues also respond to plasmid DNA (pDNA) electrotransfer, primarily by activation of cytosolic DNA sensing [3,6,7]. The central signaling complex in this pathway is the cyclic GMP-AMP synthase-stimulator of interferon genes (cGAS-STING) [8]. STING is an essential adaptor protein crucial for the inflammatory response to cytosolic DNA. Post-translational modifications (PTMs) of STING are an additional regulatory mechanism to the protein–protein interactions and the direct interaction between STING and its agonists, cyclic dinucleotides.

PTMs are generally recognized as regulatory switches that change protein function by decorating or removing proteins with molecular entities. Several PTMs have been reported to modify STING, including SUMOylation [9], deSUMOylation [9], phosphorylation [10,11,12], dephosphorylation [13], ubiquitylation [14], deubiquitylation [15], oxidation [16], carbonylation [16], alkylation [17], glycosylation [17], disulfide bond formation [18] and palmitoylation [19,20]. Some PTMs promote activation, whereas others inhibit STING activation, and these modifications are catalyzed by specific enzymes. Biologically and pathologically important PTMs such as acetylation, deacetylation, methylation, biotinylation, ribosylation and carboxylation have not yet been investigated with respect to STING.

Palmitoylation, which involves adding fatty acids to cysteine residues of proteins, is a vital post-translational modification that exerts diverse control over protein function. STING modification by palmitoylation is associated with STING polymerization to act as a platform for signal transduction [8]. Although predictable, it is still unknown if STING can be depalmitoylated.

The process of palmitoylation is orchestrated by two key players. Protein acyltransferases (PATs) or palmitoyltransferases, specifically known as zinc finger and DHHC motif containing (zDHHC) proteins, are responsible for adding palmitate to proteins. Several depalmitoylases or acyl protein thioesterases (APTs) remove palmitate, allowing the modification to be reversible [20]. These enzymes are instrumental in regulating how palmitoylation impacts protein function and trafficking [21,22].

STING has been reported as palmitoylated at the Golgi and is essential for its activation. A broad-spectrum palmitoylation inhibitor, 2-bromopalmitate (2-BP), suppressed palmitoylation and abolished type I interferon response. Mutation of Cys88/91 suppressed STING palmitoylation [19]. Selective small-molecule antagonists of STING were characterized to covalently target the predicted transmembrane cysteine residue 91 and blocked STING activation induced by palmitoylation. These compounds, as well as their derivatives, reduce inflammatory cytokine production in human and mouse cells [23]. Nitro-fatty acids were also reported to inhibit STING palmitoylation by covalent binding and inhibiting type I interferon (IFN) production in fibroblasts derived from a STING-associated vasculopathy with onset in infancy (SAVI) patient [24]. These studies showcase the importance of STING palmitoylation sites as potential targets in the treatment of STING-dependent inflammatory diseases. Recently, Chan et al. (2023) found that STING has a basal palmitoylated cysteine site (Cys64), which is required for STING activation but inaccessible to drugs [25].

Although PEFs have been extensively studied in vitro and in vivo, their effects on STING signaling through cells and tissues are yet to be fully characterized. In this study, we investigated whether STING palmitoylation and polymerization was mediated by electroporation or pDNA electrotransfer. We observed the unexpected palmitoylation of a third site on STING after the electroporation of skeletal muscle. This palmitoylation was associated with STING polymerization, which is typically associated with STING function. However, we found that the STING palmitoylation and polymerization after PEF application were not associated with STING activity. We discovered that the enzymes associated with the reversable addition and removal of palmitate are highly regulated by PEF application. Our results suggest that a decline in palmitate removal rather than an increase in palmitate addition is responsible for the PEF effects.

## 2. Materials and Methods

### 2.1. Bioinformatics

To determine whether STING was identified as a predicted or validated substrate for palmitoylation, we used the SwissPalm database (10 September 2023) [26,27]. The predicted sequence features were visualized with Protter, an open-source tool [28]. Amino acid sequences were obtained from the Uniprot database [29]. Multiple sequence alignment and homology was performed using the T-Coffee Multiple Sequence Alignment Program [30] and CLUSTALW (version 1.83) [31]. STING_MOUSE was compared by the protein sequence of the following species: *Homo sapiens* (human), *Bos taurus* (bovine), *Gallus gallus* (chicken), *Nematostella vectensis* (starlet sea anemone), *Sus scrofa* (pig), *Rattus norvegicus* (rat), *Rhinolophus ferrumequinum* (greater horseshoe bat), *Xenopus tropicalis* (western clawed frog) and *Danio rerio* (zebrafish).

To investigate the functional interaction networks of the zDHHC enzymes and cGAS-STING pathway proteins, we utilized STRING 11.0 [32]. We applied a threshold confidence level of 0.4 to identify protein interactions, and for network generation, we used seven types of protein interactions, including neighborhood, gene fusion, co-occurrence, co-expression, experimental, database knowledge and text mining.

### 2.2. Muscle Protein and RNA

This analysis used previously described animal samples [4]. All procedures were approved by the University of South Florida Institutional Animal Care and Use Committee (protocol R IS00007249, 2019). Briefly, 50 μL of an empty vector plasmid (2 mg/mL gWiz blank, Aldevron, Fargo, ND, USA) was delivered by electroporation into the right caudal thigh muscle of female 7- to 8-week C57Bl/6J mice (Jackson Laboratories, Bar Harbor, ME, USA). Eight 20-millisecond pulses at a voltage-to-distance ratio of 100 V/cm were applied using an ECM830 pulse generator using a 2-needle electrode with a 5 mm gap (BTX Harvard Apparatus, Holliston, MA, USA). For all procedures, the animals were anesthetized in an induction chamber infused with 2.5% isoflurane (Mallinckrodt Veterinary Inc., Mundelein, IL, USA) in O_2_ and fitted with a standard rodent mask supplied with the same mixture. Each mouse was monitored continuously until recovery from anesthesia. After four hours, the animals were euthanized, the muscle samples were collected and snap-frozen on dry ice. Protein and RNA purification, RNAseq and bioinformatic analysis was performed as previously described [4].

### 2.3. APEGS Assay

The acyl-PEGyl exchange gel shift (APEGS) assay was performed on muscle homogenates to confirm the palmitoylation state of STING [33]. Homogenates were suspended in buffer containing 4% sodium dodecyl sulfate (SDS), 5 mM ethylenediaminetetraacetic acid (EDTA) and Halt Protease Inhibitor Cocktail, EDTA-free (Thermo Fisher Scientific, Waltham, MA, USA). After sonication and centrifugation at 10,000× *g* for 15 min, supernatant proteins (0.4 mg/mL, 150–200× *g*) were reduced with 25 mM tris(2-carboxyethyl)phosphine for 1 h at room temperature (RT), and free cysteine residues were alkylated with 50 mM N-ethylmaleimide (NEM, N-ethylmaleimide, Thermo Fisher Scientific, Waltham, MA, USA) for 3 h at RT to be blocked. After chloroform/methanol precipitation (CM ppt), samples were suspended in PBS with 4% SDS and 5 mM EDTA then incubated in buffer (1% SDS, 5 mM 130 EDTA, 1 M NH2OH (hydroxylamine), pH 7.0) for 1 h at RT to cleave palmitoylation thioester bonds.. As a negative control, 1 M Tris-HCl, pH7.0, was used. After CM ppt, the resuspended proteins (0.5 mg/mL, 50 μg) in PBS with 4% SDS were covalently conjugated with polyethylene glycol (PEGylated) with 10 mM, 20 mM and 40 mM methoxy polyethylene glycol (mPEG, 5 k, NOF America, White Plains, NY, USA) for 1 h at RT to label newly exposed cysteinyl thiols. As a negative control, 20 mM NEM was used instead of mPEG. After CM ppt, protein precipitates were resuspended with SDS-sample buffer and boiled at 95 °C for 3 min. A BCA protein assay in individual steps measured protein concentration for normalization. A Western blot using protein-specific antibodies (described below) was performed to visualize the molecular weight shift.

### 2.4. Cell Culture

C2C12 murine myoblast cells (CRL-1772, American Type Culture Collection, Manassas, VA, USA) were cultured under sterile conditions in Dulbecco’s modified Eagle medium (DMEM, Corning, Manassas, VA, USA) supplemented with 5% fetal bovine serum (FBS, Atlanta Biologicals, Flowery Branch, GA, USA) and 1× Penicillin-Streptomycin (Gibco, Grand Island, NY, USA) at 37 °C in 5% CO_2_. The cells were repeatedly tested and found negative for mycoplasma infection using the Myco-Sniff PCR Detection Kit (MP Biochemicals, Irvine, CA, USA).

### 2.5. Cell Transfection

A plasmid-encoding firefly luciferase, gWiz Luc (Aldevron), was suspended in sterile physiological saline. Endotoxin levels were confirmed to be <100 endotoxin units/mg by the manufacturer. The myoblasts were suspended 2.0 × 10^7^ cells/mL in electroporation buffer [34] containing 0.4 μg/μL pDNA. This mixture was transferred between two stainless-steel-plate electrodes with a 2 mm gap (a kind gift of Maja Cemazar, Institute of Oncology Ljubljana, Slovenia) or into a cuvette (BTX Harvard Apparatus, Holliston, MA, USA). Six 100 s pulses at a voltage-to-distance ratio of 1300 V/cm and a frequency of 4 Hz were applied [3]. A recent study demonstrated high cell viability is maintained for up to 4 days after electroporation using this pulse protocol [35]. The cells were transferred into 6-well culture plates containing FBS. DMEM medium was added after 5 min, and the cells were incubated at 37 °C in 5% CO_2_ for the time specified in each experiment.

### 2.6. Protein Analyses

For Western blots, total protein was extracted from cell and tissue samples using Mammalian Lysis Buffer (Sigma-Aldrich, St. Louis, MO, USA) mixed with Halt™ Protease Inhibitor Cocktail (100×) (Thermo Fisher Scientific, Waltham, MA, USA). The concentration of protein was quantified using a Pierce™ BCA Protein Assay Kit (Thermo Fisher Scientific). After mixing with 4× Laemmli Sample Buffer (Bio Rad, Hercules, CA, USA) denaturation at 95 °C for 5 min, protein samples were loaded, subjected to 10% sodium dodecyl sulfate (SDS) or native polyacrylamide gel electrophoresis (PAGE). Total protein was quantified using the 2,2,2-trichloroethanol method [36] using a Chemidoc MP Imaging System (Bio-Rad, Hercules, CA, USA). For Ponceau S quantification of the total proteins in native gels, nitrocellulose membranes were stained for 5 min in 0.1% Ponceau S in 5% acetic acid (Sigma-Aldrich), then visualized using a Chemidoc MP Imaging System (Bio-Rad). Proteins were transferred to 0.45 µm nitrocellulose membranes (Bio Rad, Hercules, CA, USA). After being blocked by 3% bovine serum albumin dissolved in 1× Tris Buffered Saline (TBS) for 1 h, these membranes were incubated with primary antibody at 4 °C overnight. Polyclonal antibody information and dilution ratio are as follows: TMEM173/STING (19851-1-AP, Proteintech, Rosemont, IL, USA) at 1:500 dilution, zDHHC1 (PA5-113194, Thermo Fisher Scientific) at 1:1000 dilution, zDHHC7 (PA5-116795, Thermo Fisher Scientific) at 1:1000 dilution, zDHHC21 (PA5-25096, Thermo Fisher Scientific) at 1:1000 dilution and PPT2 (15429-1-AP, Proteintech, Rosemont, IL, USA) at 1:500 dilution. The nitrocellulose membranes were washed in TBS containing Triton X-100 (TBST) buffer and incubated with goat anti-mouse IgG antibody, (H + L) HRP conjugate (AP308P, Sigma-Aldrich) at 1:10,000 dilution for 1 h at room temperature. After washing 4 times with TBST for 5 min, proteins were visualized with Clarity Western ECL Substrate (Bio-Rad, Hercules, CA, USA), and the membranes were visualized and imaged using a Chemidoc MP Imaging System (Bio-Rad). The data analysis was performed using ImageLab software 6.1 (Bio-Rad) and ImageJ software, version 1.54h 15 December 2023 [37].

IFN-β protein levels in cell-culture supernatants were quantified by ELISA (Mouse IFN-Beta ELISA Kit, High Sensitivity, PBL Assay Science, Piscataway, NJ, USA).

### 2.7. Cell Viability and Luciferase Expression Levels

Cell viability was quantified by resazurin reduction (PrestoBlue, Invitrogen, Thermo Fisher Scientific) 4 h after transfection as per the manufacturer’s instructions. Luciferase expression was quantified following a previously described protocol [6].

### 2.8. Inhibition of STING Signaling

STING signaling in myoblasts was inhibited by incubation with 1 µM STING palmitoylation inhibitor H-151 (Cayman Chemical, Ann Arbor, MI, USA) for 1 h, as described previously [38]. The broad-spectrum palmitoylation inhibitor 2-bromopalmitate (2-BP, Sigma-Aldrich) was tested by incubation at 25 and 50 µM for 1 h, as described previously [19].

### 2.9. Statistical Analyses

To carry out statistical evaluation of the differences between groups and graph preparation, we used GraphPad Prism 9.1.0 (San Diego, CA, USA). Since the data were normally distributed, significance was determined by a one-way ANOVA test followed by a Tukey–Kramer post-test. A *p*-value of less than 0.05 was considered statistically significant.

## 3. Results

### 3.1. Pulsed Electric-Field Application in Mouse Skeletal Muscle Induces STING Palmitoylation

**Figure 1:**
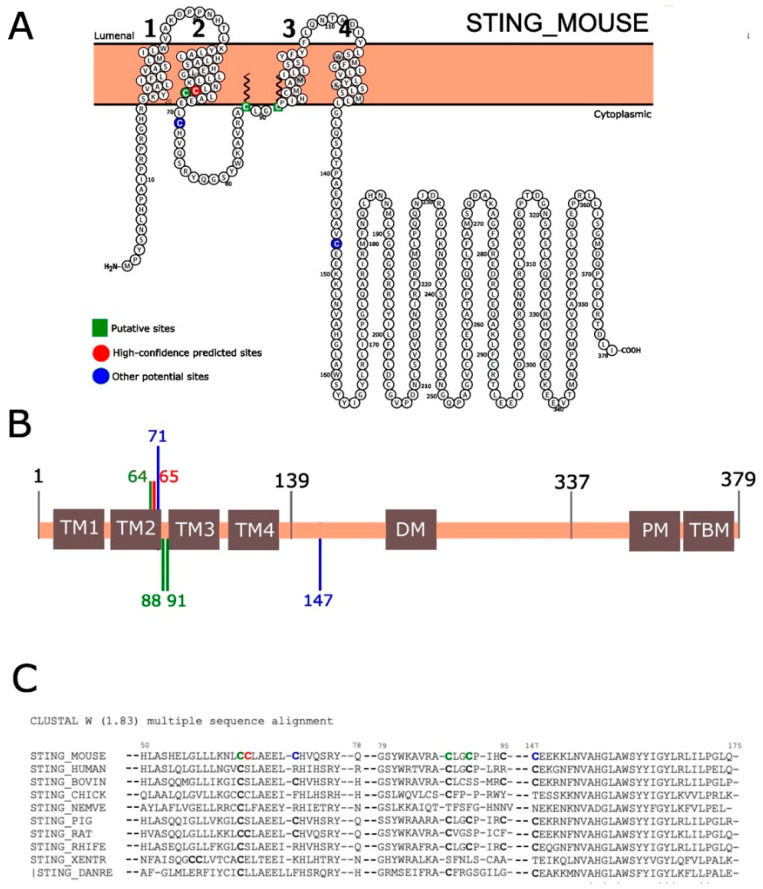
Mouse STING organization domains and palmitoylation of STING. (**A**) The major high confidence predicted cysteine palmitoylation sites in the STING_MOUSE protein amino acid (aa) sequences are represented in red, the putative palmitoylation sites are represented in green and other potentially modified sites are represented in blue. (**B**) Domain architecture representation: 1–139 aa, N-terminal domain; 139–337 aa, ligand-binding domain; 337–379 aa, C-terminal tail. TM1, TM2, TM3 and TM4 are transmembrane domains. DM, dimerization motif. PM, phosphorylation motif. TBM, TBK1-binding motif. (**C**) Sequence alignment of STING from *Mus musculus* (STING_MOUSE), *Homo sapiens* (STING_HUMAN), *Bos taurus* (STING_BOVIN), *Gallus gallus* (STING_CHICK), *Nematostella vectensis* (STING_NEMVE), Sus scrofa (STING_PIG), Rattus norvegicus (STING_RAT), *Rhinolophus ferrumequinum* (STING_RHIFE), *Xenopus tropicalis* (STING_XENTR), *Danio rerio* (|STING_DANRE). Identical cysteine residues in all sequences are indicated in bold. Mouse putative sites are indicated in green, mouse high-confidence predicted sites are indicated in red and other potential palmitoylated sites are indicated in blue.

We studied the palmitoylation of STING in the skeletal muscles of mice to understand how its activation could be influenced after PEF application or pDNA electrotransfer. Using the SwissPalm database, we predicted the palmitoylation sites on STING with high confidence (Figure 1A,B). The analysis suggested three palmitoylation sites in STING, which we confirmed through protein sequence alignment. The alignment showed that the cysteine residues are conserved across species (Figure 1C), indicating their importance in palmitoylation. These findings confirm that protein palmitoylation plays a crucial role in the evolution of STING [19,20].

**Figure 2:**
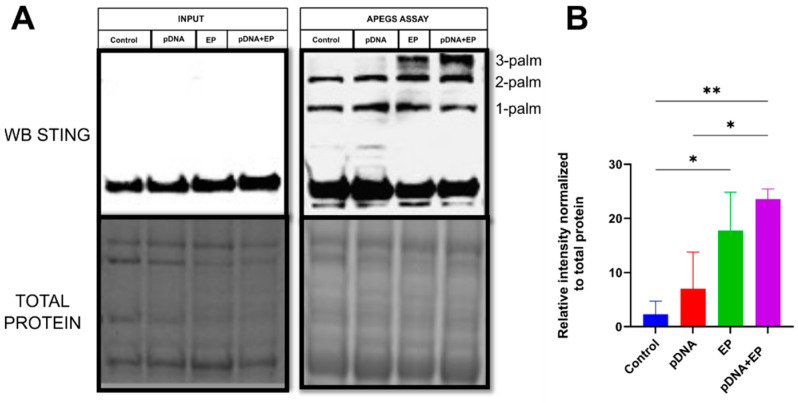
Quantification of in vivo STING palmitoylation stoichiometry using the APEGS assay. (**A**) Control STING Western blot (WB) of total protein muscle homogenate (upper right panel, Input), STING WB with APEGS assay (lower left panel), total protein gel visualization (bottom panels). 1-palm-, 2-palm-, 3-palm-, palmitoylation. (**B**) STING palmitoylation stoichiometry was determined by measuring the relative band intensity of the additional palmitoylation state (3-palm, tripalmitoylated) in the Western blot. ** *p* < 0.01, * *p* < 0.05 with respect to the control, *n* = 3. Control, naïve muscle; EP, PEF application; pDNA, gWiz Blank; pDNA+EP, pDNA electrotransfer.

To characterize the effects of the pDNA electrotransfer of the mouse muscle on STING palmitoylation modifications, we used a modified acyl-biotin exchange procedure termed the APEGS assay to identify palmitoylation sites on specific proteins [39]. The APEGS assay effectively tags the palmitoylation sites of protein substrates with a PEG polymer, causing a molecular-weight-dependent gel shift in immunoblot analyses. Thus, we quantitatively analyzed STING palmitoylation in mouse skeletal muscle homogenates. The APEGS assay precisely detected two palmitoylation sites represented by mobility shifts in the control and pDNA-injection groups (Figure 2A). Interestingly, one additional gel-shift band was detected after pulse application only (*p* < 0.05) and pDNA electrotransfer (*p* < 0.01), indicating significantly increased STING palmitoylation occurred following electroporation independently of pDNA injection (Figure 2B).

### 3.2. Pulsed Electric-Field Application in Mouse Skeletal Muscle Induces STING Polymerization

**Figure 3:**
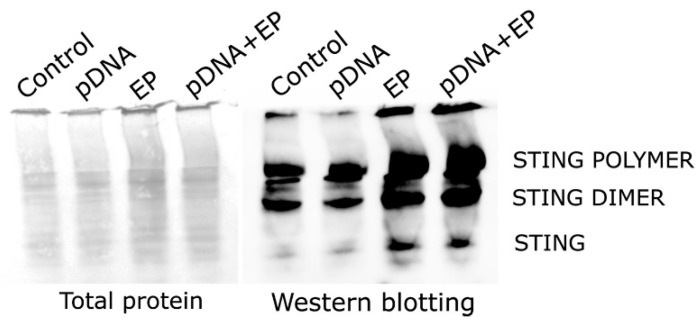
STING polymerization in myoblasts 4 hours after pDNA electrotransfer of myoblasts. Ponceau S staining was performed on native PAGE nitrocellulose membranes. Left panel, total protein; right panel, Western blot. Control, naïve cells; EP, pulse application; pDNA, gWiz Luc; pDNA+EP, pDNA electrotransfer.

Previous research demonstrated that human STING ligand binding leads to polymerization [18]. However, it was still uncertain whether pulsed electric fields cause STING polymerization in mouse myoblasts. Native PAGE revealed that STING polymerization occurred independently of ligand binding or the presence of DNA (Figure 3). This indicated that electroporation triggered STING polymerization and subsequent palmitoylation.

### 3.3. IFN-β Expression Induced by pDNA Electrotransfer Reduced by STING and Palmitoylation Inhibitors

**Figure 4:**
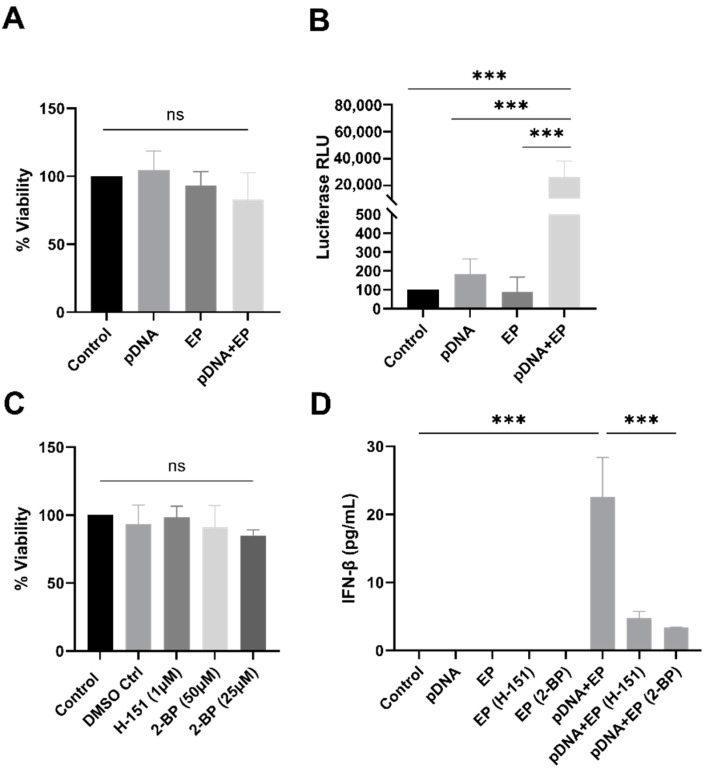
Effects of STING activation and inhibition on STING signaling 4 hours after pDNA electrotransfer of myoblasts. (**A**) Viability as indicated by cell metabolism normalized to the control cells. (**B**) Luciferase expression. (**C**) Effect of inhibitors H-151 and 2-BP on viability. (**D**) Effect of inhibitors H-151 and 2-BP on IFN-β secretion. *** *p* < 0.001 with respect to the control, *n* = 3, ns = non-significant. Control, naïve cells; EP, pulse application; pDNA, gWiz Luc; pDNA+EP, pDNA electrotransfer. DMSO, dimethyl sulfoxide.

Using RNA sequencing, we previously compared the gene and protein expression of control skeletal muscle to groups that received a pDNA injection, pulse application and pDNA electrotransfer four hours after delivery [4]. Our analysis revealed that several pathways, including the chemokine-signaling pathway, were highly enriched in each experimental group with respect to naïve muscle. The present study focused on type I IFN, a biomarker of cGAS-STING-generated proinflammatory signaling. We first confirmed that electrotransfer did not impact cell viability (Figure 4A) and that, as expected, pDNA electrotransfer significantly (*p* < 0.001) increased transfection levels as indicated by luciferase activity (Figure 4B). We next confirmed that exposure to the STING palmitoylation inhibitor H-151 did not reduce cell viability (Figure 4C). Despite the known toxicity of 2-BP, the increasing concentrations of this broad-spectrum palmitoylation inhibitor did not reduce myoblast viability (Figure 4C). Finally, we verified that STING palmitoylation is critical for IFN-β production. Palmitoylation inhibition significantly reduced STING signaling (Figure 4D) in the pDNA electrotransfer group (*p* < 0.001). Our findings confirmed previous reports [19,20] that STING palmitoylation is crucial for inducing IFN-β secretion.

### 3.4. Pulsed Electric Fields Induce Changes in Palmitoyltransferase Expression

**Figure 5:**
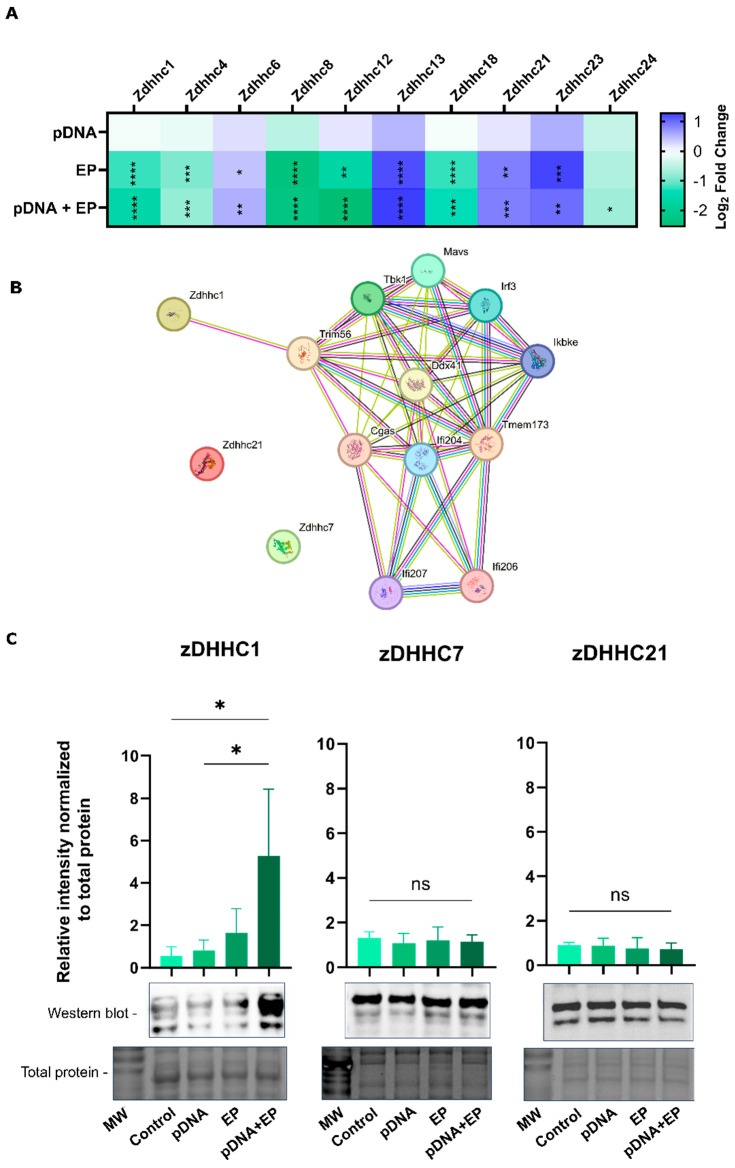
Expression of palmitoyltransferases in skeletal muscle and myoblasts. (**A**) Heatmap reveals the expression of genes that palmitoylate proteins in mouse skeletal muscle 4 hours after pDNA delivery compared to control muscle, *n* = 4–5 per group. (**B**) STRING database analysis of protein networks related to cytosolic DNA sensing, Tmem173, STING. (**C**) Western blots showing the effect of pDNA electrotransfer on zDHHC1, zDHHC7 and zDHHC21 protein levels in C2C12 myoblasts, *n* = 3. **** *p* < 0.0001, *** *p* < 0.001, ** *p* < 0.01, * *p* < 0.05 compared to the control. Control, naïve cells or tissues; EP, pulse application; pDNA, gWiz Luc; pDNA+EP, pDNA electrotransfer.

Cytosolic DNA sensing must be regulated accurately to avoid an excessive innate immune response. Analysis of skeletal muscle RNAseq data revealed the expression of PAT mRNAs (Figure 5A). We found that PAT gene expression after pDNA injection did not vary from control tissue. While a group of genes, zDHHC6, zDHHC13, zDHHC21 and zDHHC23, were upregulated in both groups receiving pulses, the expression of a number of the genes, zDHHC1, zDHHC4, zDHHC8, zDHHC12 and zDHHC18, was downregulated in both pulsed groups. The expression of one PAT, zDHHC24, was downregulated after pDNA electrotransfer only. 

The STRING database was used to query protein interactors of cGAS-STING signaling. In this network, each node represents a protein, while the edges between them indicate the predicted functional associations. An edge may have up to four different colored lines, each one representing a different type of evidence for the predicted association. A green line is for neighborhood evidence, a blue line is for cooccurrence evidence, a purple line is for experimental evidence, a yellow line is for text-mining evidence and a black line is for co-expression evidence. A protein–protein interaction network analysis through this database identified a loose network of proteins containing a single zDHHC protein, zDHHC1; zDHHC7 and zDHHC21 are shown but not directly interacting with the network (Figure 5B).

Although the zDDHC1 gene expression was downregulated by pulse application (Figure 5A), this protein was highly connected with clusters around the cGAS-STING pathway (Figure 5B). Western blots (Figure 5C) demonstrated a 5-fold upregulation of zDHHC1 after pDNA electrotransfer (*p* < 0.05). We also quantified zDHHC7 and zDHHC21 proteins; no changes in expression were observed.

### 3.5. Pulsed Electric Fields Induce Changes in Depalmitoylase Expression

**Figure 6:**
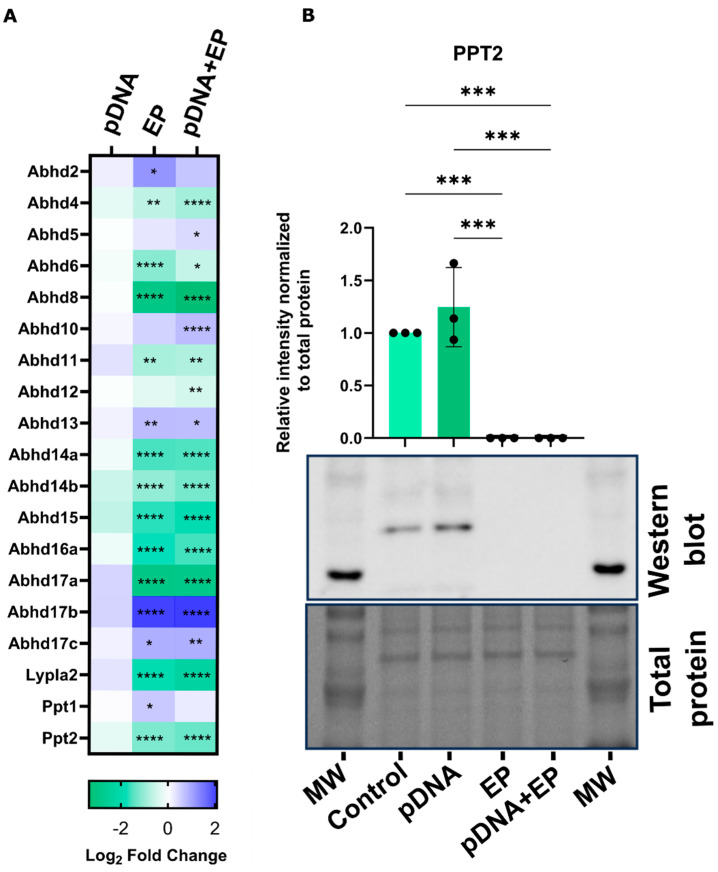
Expression of depalmitoylases in skeletal muscle and myoblasts. (**A**) Heatmap reveals depalmitoylase gene expression regulation in mouse skeletal muscle 24 hours after pDNA delivery compared to control muscle, *n* = 4–5 per group. (**B**) Western blots showing the effect of pDNA electrotransfer on PPT2 protein levels in C2C12 myoblasts, *n* = 3. **** *p* < 0.0001, *** *p* < 0.001, ** *p* < 0.01, * *p* < 0.05 compared to the control. Control, naïve cells or tissues; EP, pulse application; pDNA, gWiz Luc; pDNA+EP, pDNA electrotransfer.

Analysis of RNAseq data revealed that no APTs were regulated by pDNA injection when compared to the control group (Figure 6A). A subgroup of α/β hydrolase domain-containing protein (Abhds) mRNAs were upregulated by pulse application, whether alone or in combination with pDNA. Abhd2 and palmitoyl-protein thioesterase 1 (Ppt1) gene expression was upregulated only in the group receiving pulses, while Abhd5 and Abhd10 were upregulated only by pDNA electrotransfer. A majority of depalmitoylase genes were significantly downregulated in both the pulsed groups as compared to control muscle. 

Western blots were performed to determine if the protein expression levels of the depalmitoylase PPT2 mirrored gene expression in myoblasts. Twenty-four hours after pDNA electrotransfer, the levels of PPT2 protein were significantly depleted in the groups receiving electroporation only and pDNA electrotransfer (Figure 6B).

This mRNA and protein downregulation in both pulsed groups may explain the increase in STING palmitoylation (Figure 2). Therefore, it is speculated that PPT2 alone or potentially combined with other APTs plays a critical role in STING palmitoylation in mouse muscle.

## 4. Discussion

In this study, we investigated whether STING palmitoylation and polymerization are mediated by pDNA electrotransfer. We performed RNA sequencing analysis in muscle tissue to identify specific signatures of DNA electroporation and investigated the mechanism of STING palmitoylation. Additionally, we conducted cell-based assays to gather information on the molecular changes in myoblasts. Our findings showed that pDNA electrotransfer activated a STING-mediated IFN-β response, but palmitoylation and polymerization were independent of the presence of pDNA. We observed that zDHHC1 was upregulated only when pDNA was electroporated and was followed by IFN-β expression, suggesting zDHHC1 as a candidate to promote STING palmitoylation, stability and activity. We also identified one additional palmitoylated STING form on mouse muscle groups that received PEF application alone. Unexpectedly, these results correlated with the downregulation of APT genes and abolishment of PPT2 protein expression on these same groups, suggesting an increase in palmitoylation as an effect of the orchestrated action of the APTs.

Palmitoylation, the addition of palmitate fatty acids to proteins, is a key post-translational modification influencing various aspects of protein function. Protein S-palmitoylation, a reversible post-translational modification, plays a pivotal role in regulating diverse biological processes. Existing literature underscores its impact on protein localization, activity, stability and interactions, with a notable role in regulating protein trafficking and membrane interactions [40]. The dynamic control afforded by the reversibility of palmitoylation is critical for the nuanced regulation of cellular processes, including signal transduction and cellular differentiation [41].

Several approaches were previously used to characterize and validate STING palmitoylation, such as 2-BP inhibition, hydroxylamine cleavage of thioester bonds, PAT localization by fluorescent tag, PAT overexpression, point mutation, substrate localization by fluorescence tag and [3H] palmitic acid labeling [19,23,24]. These validation experiments were essential for the discovery of mouse STING palmitoylation sites, but most of them, as well as current acyl-biotinyl exchange (ABE) chemistry or clickable probes, do not measure in vivo palmitoylation stoichiometries effectively. These techniques were performed in mouse embryonic fibroblasts, human HEK293T or monkey COS1 kidney cells and bone-marrow-derived macrophage cells. Palmitoyl-proteome experiments were also used to identify STING as a palmitoyl substrate [42,43]. These studies identified STING in the palmitoylation fraction using click chemistry in BW5147-derived mouse T-cell hybridoma and neural stem cells, respectively. The APEGS assay allows the quantification of palmitoylation levels on proteins in various biological samples including tissue samples [33]. Our study confirmed the presence of three distinct palmitoylation sites on the STING protein (Figure 2). Two sites were constitutively palmitoylated in all groups. Interestingly, palmitoylation of the third site was confirmed by an APEGS assay not only in response to intercellular DNA entry as expected after pDNA electrotransfer but also after PEF application alone.

Activation of STING produces protein complexes that can be resolved as monomers, dimers and polymers with higher molecular weights. As a result of intermolecular disulfide linkages, higher-molecular-weight STING polymers are formed [18]. These polymers have also been referred to as oligomers or aggregates [23,44,45,46,47,48,49]. STING polymers are considered active [18,45]. STING dimers and polymers were visualized in non-reducing gel conditions, indicating that disulfide bonds form all STING dimers and polymers. These dimers and polymers were detected not only in response to intercellular DNA entry by pDNA electrotransfer but also after electroporation alone (Figure 3). However, we observed STING polymerization after PEF application did not correlate with STING activation as indicated by IFN-β production (Figure 4).

Our study delved into the regulatory mechanisms of STING activation and its impact on type I interferon signaling, specifically palmitoylation. To assess the role of palmitoylation in IFN response, we employed STING inhibitor H-151 and the palmitoylation inhibitor 2-BP in transfected myoblasts. Our findings demonstrate a significant reduction in IFN-β secretion, confirming the crucial involvement of STING palmitoylation in inducing an immune response after pDNA electrotransfer (Figure 4).

An active upregulation of palmitoylases in response to PEF application might contribute to an additional site for STING palmitoylation. The STRING analysis suggests a correlation between zDHHC1 and palmitoylation (Figure 5B). In particular, zDHHC1, identified as an ER-associated protein, positively regulates virus-triggered STING-dependent immune signaling [50]. The interaction between zDHHC1 and interferon-induced transmembrane protein 3 (IFITM3) could be the driver of palmitoylation and stability of zDHHC1. Knockdown of IFITM3 weakens the inhibitory role of zDHHC1 on virus replication [51]. Without zDHHC1, cells exhibit a reduced ability to produce IFN-β and other cytokines in response to DNA viruses. In Zdhhc1(−/−) mice infected with HSV-1, lower cytokine levels in the brain result in increased lethality. zDHHC1 interacts with STING, facilitating the dimerization or polymerization of STING and recruitment of downstream signaling components TBK1 and IRF3 [50]. Interestingly, zDHHC1 mRNA was significantly downregulated in each group in which pulses were applied (Figure 5A). However, protein levels were upregulated only during pDNA electrotransfer (Figure 5B). It is well-established that protein levels do not necessarily reflect changes in gene expression [52,53]. The lack of protein regulation reduced the likelihood that zDHHC1 was responsible for the increase in palmitoylation observed after PEF application only.

Depalmitoylase gene expression is also highly regulated in response to electroporation. The gene expression of twelve depalmitoylases was reduced (Figure 6A). A nearly complete reduction was confirmed by the examination of the APT PPT2 protein (Figure 6B). This observation indicates that PPT2 downregulation may be responsible for the coordinate upregulation of STING palmitoylation. However, palmitate was not hydrolyzed from several palmitoylated protein substrates by PPT2 [54]. The effect of PPT2 downregulation on STING palmitoylation may be related to acyl CoA metabolism. RNA blot hybridization analysis of human tissues revealed notably elevated PPT2 expression in muscle [54].Therefore, the specific relationship between STING and PPT2 may be limited or may be a skeletal-muscle-specific phenomenon. While these observations support this study, STING palmitoylation and polymerization may not be universally induced after the application of other pulse regimens.

Our investigation builds upon a wealth of previous research focused on assessing the impact of pulse application to mammalian cells. These observations include protein modifications such as phosphorylation in Jurkat T lymphocytes and HeLa adenocarcinoma cells [55,56,57,58]. PEF application induces aggregation of the inflammasome adaptor protein ASC in multiple innate immune cell types [59]. In myoblasts and skeletal muscle, transient changes in endogenous gene and protein expression in response to PEF application alone have been described [1,2,3,4,5].

These findings reveal a layer of complexity in STING regulation. The physical stimulus of PEF application raises intriguing questions about the physical properties driving this effect. Potential factors such as heat, pH changes or reactive oxygen species warrant further investigation. Several other regulatory mechanisms influencing STING activation are documented. Mutations and alkylation of specific cysteine residues impair type I IFN response induction, and glutathione peroxidase inactivation hinders STING trafficking, suppressing its localization to the Golgi complex [19]. Interestingly, RNA expression associated with glutathione peroxidase activity is regulated after PEF application and pDNA electrotransfer [5], supporting the theory that STING trafficking may be affected. These insights underscore the multifaceted nature of STING regulation, providing a foundation for further exploration and understanding in the context of our study. Overall, these data confirm that PEF application induced the production of disulfide-bonded STING polymers (Figure 3), which coincided with the triad-palmitoylated STING forms found in muscle-receiving pulses (Figure 2). Therefore, our results align with the notion that post-translational modifications, such as palmitoylation, may play a crucial role in modulating STING activity in response to PEF application. Contrary to the plausible expectation that palmitoyltransferase upregulation would drive palmitoylation of the third STING site, our findings suggest that the increase in palmitoylation is more likely a consequence of depalmitoylase downregulation.

## 5. Conclusions

Our study discovered the regulatory mechanisms of STING palmitoylation in mouse skeletal muscle in response to PEF application. We identified three potential palmitoylation sites on STING and confirmed increased palmitoylation post-electroporation. STING palmitoylation plays a crucial role in immune response modulation, as highlighted by the effectiveness of STING inhibitors in reducing IFN-β secretion. Our study provides valuable insights into the complex regulation of STING in the context of palmitoylation. In conclusion, our study contributes valuable insights into the effects of PEFs on the intricate network of regulatory pathways governing STING activation and type I IFN signaling.

## Data Availability

The data presented in this study are available on request from the corresponding author.
